# Supplementary material for: Oncogenic ERRB2 signals through the AP-1 transcription factor to control mesenchymal-like properties of oesophageal adenocarcinoma
Source: NAR Cancer. 2023 Jan 23;5(1):zcad001. doi: 10.1093/narcan/zcad001 (PMC9869078; doi:10.1093/narcan/zcad001)
Supplement: zcad001_Supplemental_Files [file zcad001_supplemental_files.zip › Supplementary Figures_revised.pdf]

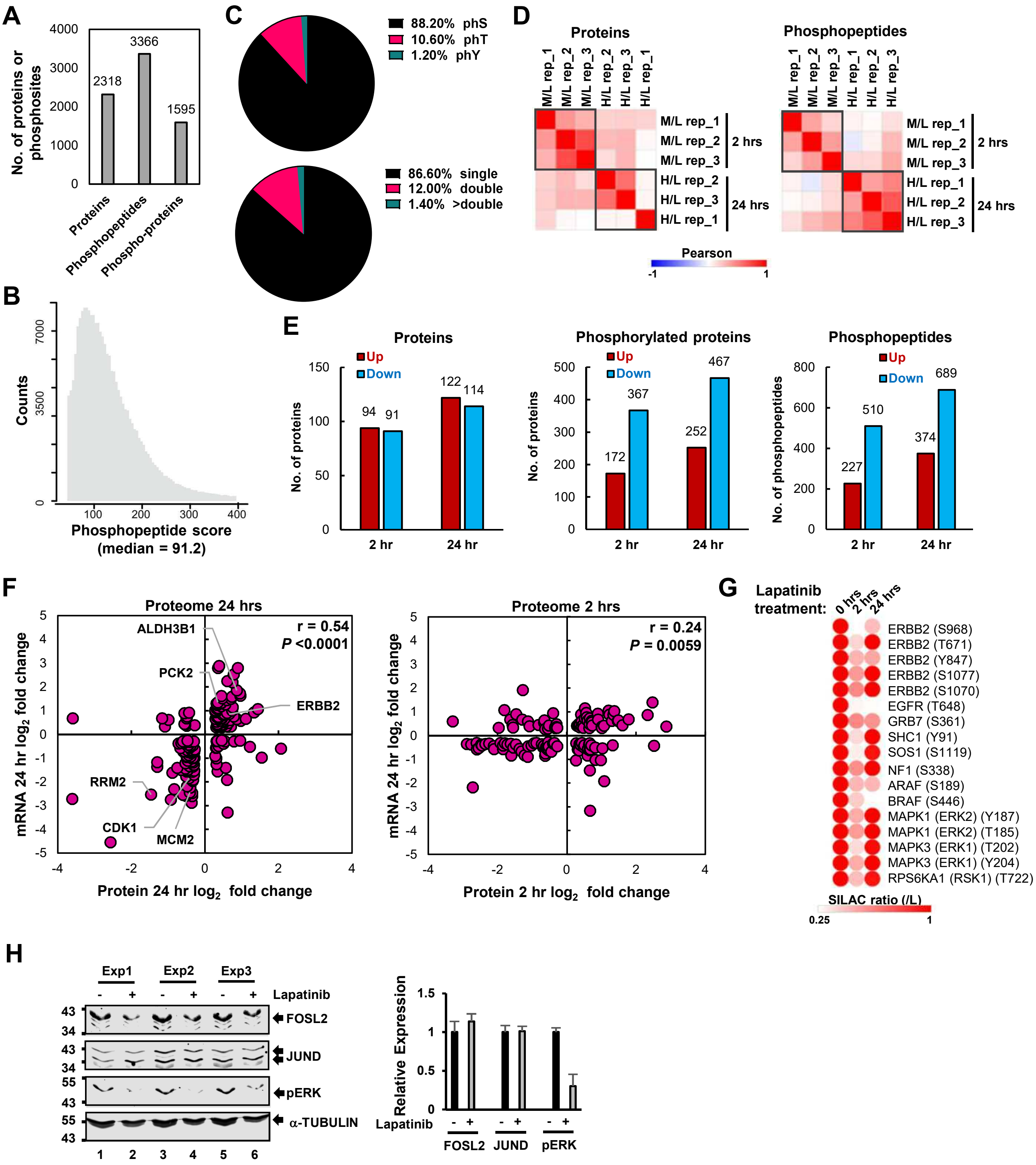

Figure S1. Phosphoproteomic analysis of ERBB2 signalling in OAC cells.
(A) The number of proteins, phosphopeptides and phosphorylated proteins detected in all 3 biological replicates across all conditions. (B) Phosphorylated peptide score. (C) Distribution of phosphorylated sites; phS – phosphoserine, phT – phosphothreonine, phY – phosphotyrosine (top) and of phosphopeptides with 1, 2 or more phosphorylated sites (bottom). (D) Pearson correlation of proteome replicates (PRO) and phosphoproteome replicates (STY) of OE19 cells treated with 500 nM lapatinib for 2 hours or 24 hours. SILAC ratios are expressed as lapatinib treated sample relative to DMSO control. H – heavy, 24 hours lapatinib; M – medium, 2 hours lapatinib; L – light, 2 hours DMSO. (E) The number of up- or down-regulated: proteins or phosphorylated proteins or phosphopeptides after 2 or 24 hours lapatinib treatment relative to DMSO control. Up- or down-regulated proteins/phosphorylated proteins/phosphopeptides were defined based upon all 3 biological replicates having a 1.2X linear fold change in the same direction. (F) Correlation of changes in the proteome after either 24 hrs (left) or 2 hrs (right) and transcriptome after 24 hours 500 nM lapatinib treatment relative to DMSO control. Only genes in which the protein and mRNA fold change was greater than 1.2 fold are shown. Pearson correlation (r) is shown. (G) Heatmap of SILAC ratios for peptides containing the indicated phosphorylation sites before (0 hr) and after (2 and 24 hr) lapatinib treatment (relative to 0 hrs). (H) Western blot of FOSL2 and JUND expression following lapatinib treatment for 24 hr. The data from three experiments (Exp1-3) are shown and quantified on the right. Phosphorylated ERK (pERK) is shown as a control.

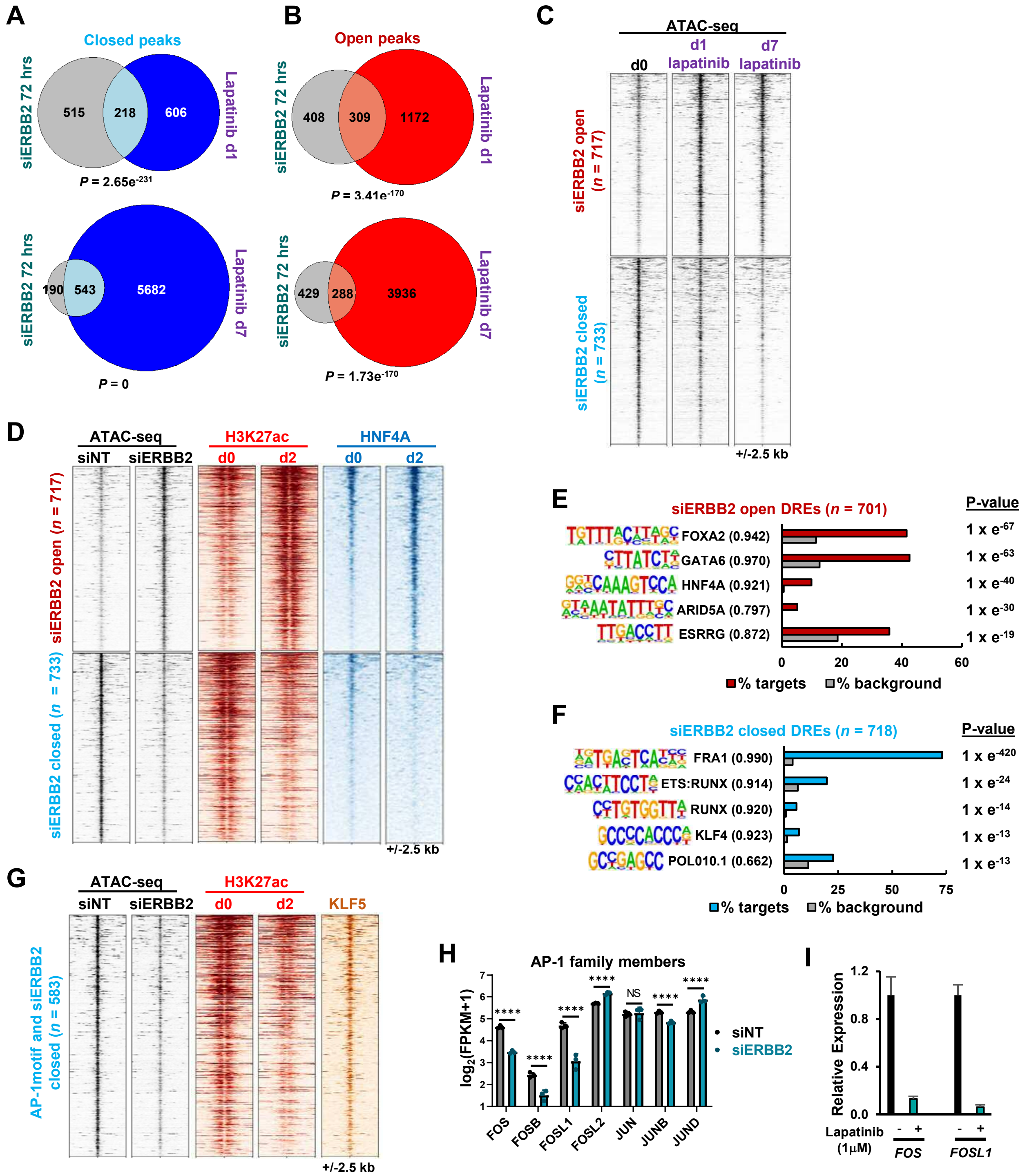

Figure S2. ERBB2 regulates AP-1 activity in ERBB2 positive OAC cells.

(A and B) Overlap of differentially accessible peaks in OE19 cells after ERBB2 knockdown (siERBB2) and 1 (d1) or 7 (d7) day 500 nM lapatinib treatment. (A) Overlap of closed peaks. (B) Overlap of open peaks. (C) Heatmap showing ATAC-seq and signal in OE19 cells treated with lapatinib (d0-d7) at differentially open or closed ATAC-seq peaks in OE19 cells following ERBB2 knockdown (siERBB2). (D) Heatmap of differentially open or closed ATAC-seq peaks in OE19 cells following ERBB2 knockdown (siERBB2). ATAC-seq is shown for OE19 cells treated with siERBB2. HNF4A ChIP-seq and H3K27ac ChIP-seq data is shown for OE19 cells treated with lapatinib for the indicated timepoints (d0 or d2). (E and F) *De novo* transcription factor motif enrichment at differentially (E) open or (F) closed distal regulatory elements in OE19 cells following *ERBB2* knockdown. Motif match score to called transcription factor is shown in brackets. (G) Heatmap of differentially closed chromatin regions containing the AP-1 motif in OE19 cells following *ERBB2* knockdown. ATAC-seq is shown for OE19 cells treated with siERBB2. H3K27ac ChIP-seq is shown for OE19 cells treated with lapatinib for the indicated timepoints (d0 or d2). Parental KLF5 ChIP-seq data is also shown. (H) mRNA expression of the indicated AP-1 subunits from RNA-seq of OE19 cells treated with siERBB2. (I) RT-qPCR analysis of *FOS* and *FOSL1* expression in the OAC organoid WTSI-OESO_009 following treatment with lapatinib for 24 hr (n=2).

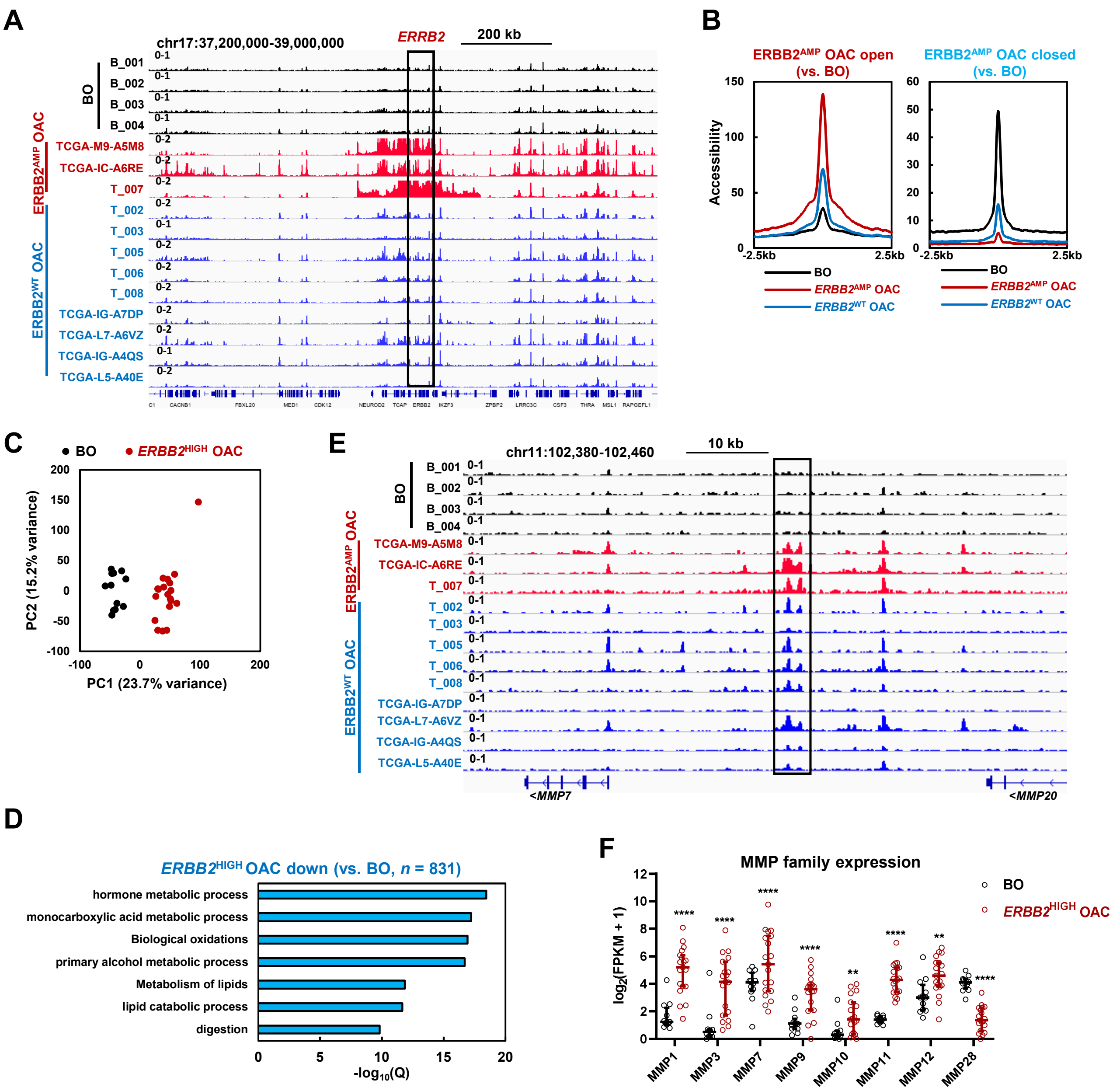

G

*ERBB2*HIGH OAC down (vs. BO)

Gene set	NES	FDR
HALLMARK_XENOBIOTIC_METABOLISM	-2.3	0.001
HALLMARK_FATTY_ACID_METABOLISM	-2.01	0.01
HALLMARK_BILE_ACID_METABOLISM	-1.92	0.016
HALLMARK_ESTROGEN_RESPONSE_LATE	-1.82	0.026

*ERBB2*HIGH OAC up (vs. BO)

Gene set	NES	FDR
HALLMARK_G2M_CHECKPOINT	3.5	0.000
HALLMARK_E2F_TARGETS	3.36	0.000
HALLMARK_TNFA_SIGNALING_VIA_NFKB	2.73	0.000
HALLMARK_MITOTIC_SPINDLE	2.37	0.000
HALLMARK_MYC_TARGETS_V2	2.11	0.004
HALLMARK_EPITHELIAL_MESENCHYMAL_TRANSITION	2.05	0.005
HALLMARK_DNA_REPAIR	1.94	0.008
HALLMARK_MYC_TARGETS_V1	1.83	0.016
HALLMARK_COAGULATION	1.76	0.024

Figure S3. AP-1 expression increases during the transition from Barrett's oesophagus to ERBB2 positive oesophageal adenocarcinoma.
(A) Genome browser view of ATAC-seq data highlighting the *ERBB2* locus in Barrett's oesophagus (BO) and OAC patient tissue samples. (B) Average tag density plot of chromatin accessibility measured by ATAC-seq in the indicated patient sample groups for differentially opening (left) and closing (right) regions in *ERBB2*AMP OAC samples relative to BO samples (2X linear fold change, FDR < 0.05). (C) PCA of RNA-seq data from BO and *ERBB2*HIGH OAC patient tissue samples. (D) GO analysis (Metascape) of genes down-regulated in *ERBB2*HIGH OAC relative to BO tissue. (E) Genome browser view showing ATAC-seq signal at *MMP7* and *MMP20* loci. BO, *ERBB2*AMP OAC and *ERBB2*WT OAC patient tissue samples are shown. (F) mRNA expression of the *MMP* encoding genes differentially expressed between Barrett's oesophagus (BO) and *ERBB2*HIGH OAC patient tissue; ** $P < 0.01$, **** $P < 0.0001$. All are up-regulated except for *MMP28*. (G) GSEA results of differentially expressed genes in *ERBB2*HIGH OAC tissue relative to BO tissue. NES – normalised enrichment score.

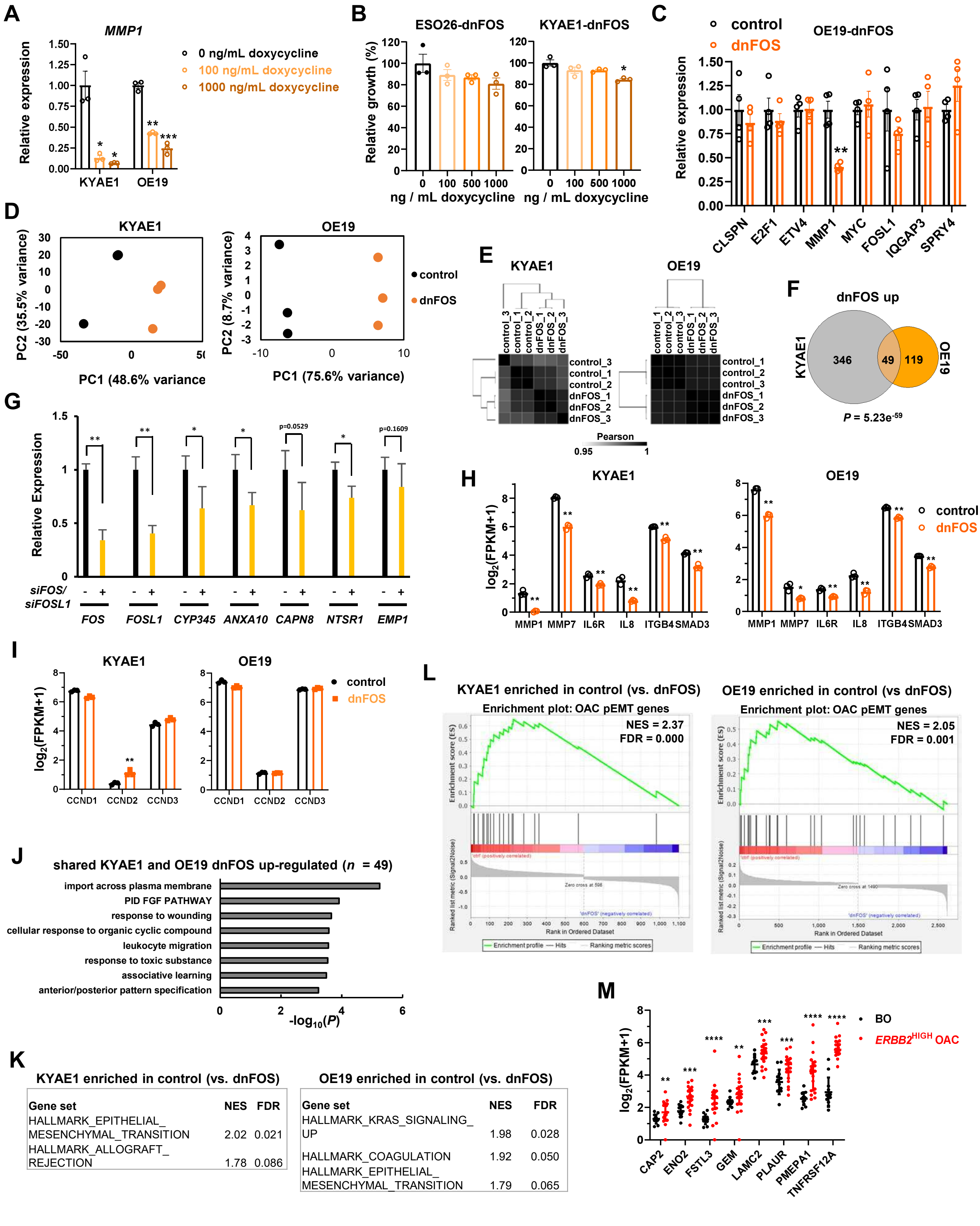

K

KYAE1 enriched in control (vs. dnFOS)

Gene set	NES	FDR
HALLMARK_EPITHELIAL_ MESENCHYMAL_TRANSITION	2.02	0.021
HALLMARK_ALLOGRAFT_ REJECTION	1.78	0.086

OE19 enriched in control (vs. dnFOS)

Gene set	NES	FDR
HALLMARK_KRAS_SIGNALING_ UP	1.98	0.028
HALLMARK_COAGULATION	1.92	0.050
HALLMARK_EPITHELIAL_ MESENCHYMAL_TRANSITION	1.79	0.065

Figure S4. AP-1 regulates processes associated with epithelial-mesenchymal transition in ERBB2 positive OAC cells. Continued overleaf….

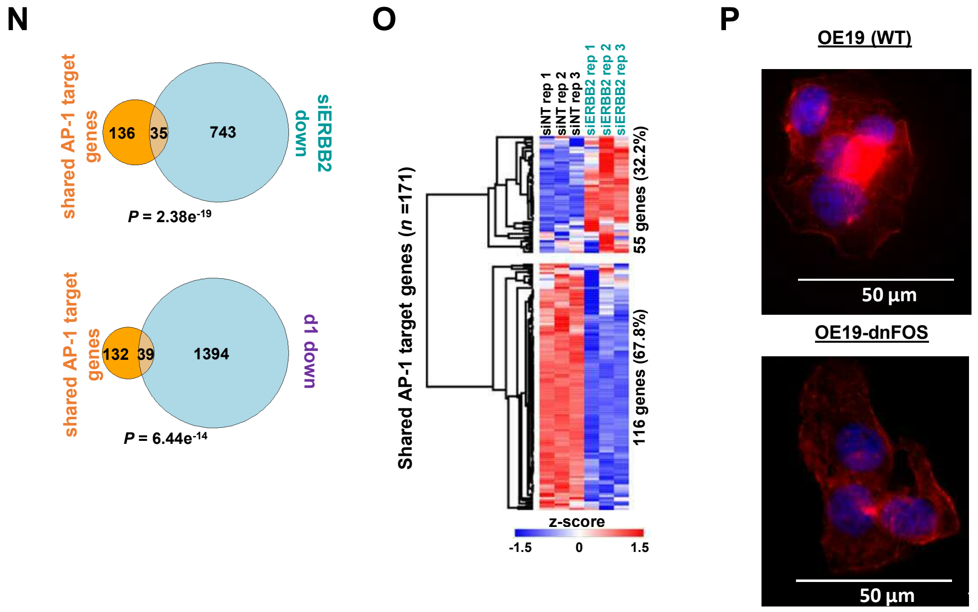

Figure S4. AP-1 regulates processes associated with epithelial-mesenchymal transition in ERBB2 positive OAC cells.
(A) RT-qPCR analysis of *MMP1* expression in KYAE1-dnFOS and OE19-dnFOS cells. GFP-dnFOS expression was induced by treatment with the indicated doses of doxycycline for 48 hours. * $P < 0.05$, ** $P < 0.01$, *** $P < 0.001$; paired T-test, $n = 3$. (B) Crystal violet growth assay of ESO26-dnFOS and KYAE1-dnFOS cells following dnFOS induction. * $P < 0.05$; paired T-test, $n = 3$. (C) RT-qPCR analysis in OE19-dnFOS cells of dnFOS down-regulated genes identified in OE33 cells (Britton et al., 2017). Control – untreated cells; dnFOS – 1000 ng / mL doxycycline for 48 hours. ** $P < 0.01$; paired T-test, $n = 4$. (D) PCA and (E) Pearson correlation analysis of KYAE1-dnFOS and OE19-dnFOS RNA-seq data ($n = 3$). Control – untreated cells; dnFOS – 1000 ng / mL doxycycline for 48 hours. (F) Overlap of genes up-regulated (0.5 $\log_2$ fold change, FDR < 0.05, FPKM > 1) by dnFOS induction in KYAE1-dnFOS and OE19-dnFOS cells. (G) RT-qPCR analysis of the expression of the indicated genes following siRNA-mediated co-depletion of *FOS* and *FOSL1* in OE19 cells (* $P < 0.05$, ** $P < 0.01$; paired T-test, $n = 3$). (H and I) mRNA expression of the indicated genes in KYAE1-dnFOS and OE19-dnFOS cells from RNAseq analysis ($n = 3$). (J) GO analysis (Metascape) of genes up-regulated by dnFOS in both KYAE1 and OE19 cells. (K) GSEA results of differentially expressed genes in KYAE1 and OE19 control cells relative to dnFOS induced cells. NES – normalised enrichment score. (L) Enrichment plot from GSEA of pEMT genes (Tyler and Tirosh, 2021) on differentially expressed up- and down- regulated genes (FDR < 0.05) in KYAE1 (left) or OE19 (right) cells following dnFOS induction. NES – normalised enrichment score. (M) Patient tissue mRNA expression of pEMT genes (Tyler and Tirosh, 2021) that are down-regulated following dnFOS induction in either KYAE1 or OE19 cells. Genes up-regulated in ERBB2^{HIGH} OAC relative to BO (FDR < 0.05) are shown. (N) Overlap of shared AP-1 target genes (genes down-regulated by dnFOS in both KYAE1 and OE19 cells) and genes down-regulated (2X linear fold change, FDR < 0.05, FPKM > 1) by siERBB2 (left) or 1 day lapatinib (right) in OE19 cells. Statistical significance was determined using a hypergeometric test. (O) Heatmap showing the z-scored expression of shared AP-1 target genes in OE19 cells treated with siERBB2. The number and percentage of up- or down- regulated genes is indicated. (P) Immunofluorescence detection of actin filaments in wild-type OE19 (WT; top) or OE19-dnFOS (bottom) cells.
